# Association between Anaemia in Children 6 to 23 Months Old and Child, Mother, Household and Feeding Indicators

**DOI:** 10.3390/nu10091269

**Published:** 2018-09-08

**Authors:** Alberto Prieto-Patron, Klazine Van der Horst, Zsuzsa V. Hutton, Patrick Detzel

**Affiliations:** 1 Nestlé Research Center, Vers-Chez-les-Blanc, Route du Jorat 57, Case Postale 44, 1000 Lausanne-26, Switzerland; alberto.prietopatron@rdls.nestle.com (A.P.-P.); zsuzsav.hutton@rdls.nestle.com (Z.V.H.); 2 Division of Nutrition and Dietetics, Department of Health Professions, Bern University of Applied Sciences, Murtenstrasse 10, CH-3008 Bern, Switzerland; klazine.vanderhorst@bfh.ch

**Keywords:** Anaemia, infancy and toddlerhood, low and middle-income countries, Demographic Health Surveys, infant feeding, multilevel regression

## Abstract

In Low and Lower-Middle-Income countries, the prevalence of anaemia in infancy remains high. In early childhood anaemia cause irreversible cognitive deficits and represents a higher risk of child mortality. The consequences of anaemia in infancy are a major barrier to overcome poverty traps. The aim of this study was to analyse, based on a multi-level approach, different factors associated with anaemia in children 6–23 months old based on recent available Standard Demographic Health Surveys (S-DHS). We identified 52 S-DHS that had complete information in all covariates of interest in our analysis between 2005 and 2015. We performed traditional logistic regressions and multilevel logistic regression analyses to study the association between haemoglobin concentrations and household, child, maternal, socio-demographic variables. In our sample, 70% of the 6–23 months-old children were anaemic. Child anaemia was strongly associated with maternal anaemia, household wealth, maternal education and low birth weight. Children fed with fortified foods, potatoes and other tubers had significantly lower rates of anaemia. Improving overall household living conditions, increasing maternal education, delaying childbearing and introducing iron rich foods at six months of age may reduce the likelihood of anaemia in toddlerhood.

## 1. Introduction

The World Health Organization (WHO) reported that in 2011 the prevalence of anaemia among 6–59 months-old children was 42.6% globally [1]. The highest prevalence rates were observed in African (62.3%), South-East-Asian (53.8%) and the Eastern-Mediterranean (48.6%) regions. The adverse health consequences of anaemia among pre-school children are well documented, some having an impact over a lifetime [2,3]. These include impaired cognitive development and growth, increased susceptibility to infections, fatigue and lower physical activity.

The social costs associated with anaemia in early childhood are vast and largely affecting children born in lower income groups. Plessow et al. estimated that over one percent of the Indian Gross Domestic Product (GDP) is lost every year due to Iron Deficiency Anaemia among 6–59-month-old children. In addition, the health burden surpassed 8 million Disability Adjusted Life Years (DALYs) and affects disproportionally younger infants from lower wealth households [4]. Over 95% of this economic and 80% of the health burden was linked to children 6 to 23 months of age.

The most frequent factors associated with anaemia among children are malnutrition and infections [5]. According to the WHO globally approximately half of the anaemia is linked to iron deficiency, although this proportion varies by geography and population groups [1,6]. Other conditions can also lead to anaemia, such as a deficiency of other micronutrients, phytate rich diets, acute and chronic infections including malaria, HIV and tuberculosis, as well as haemoglobinopathies [7,8,9].

In addition, maternal, household and community factors have been reported to increase the risk of being anaemic in early childhood. The complexity of these factors and their interaction require multi-faceted strategies to address anaemia globally. However, it is difficult to draft optimal policies without untangling the relative contribution of these factors associated with increased risk of anaemia and their inter-relation.

Anaemia among infants is determined by a large number of factors. For example, poverty per se leads to increased food insecurity and lower sanitation facilities but could only be considered as the cause of anaemia indirectly. It acts through inter-related determinants (intermediate variables). These intermediate determinants can be further divided into hierarchically or parallel inter-related sub-groups. Victora et al. proposed the use of conceptual frameworks in epidemiological multivariate analysis [10]. They postulate that multivariate analysis is most often used to determine the effect of an assumed risk factor on the investigated outcome after controlling for cofounding factors to explore whether the effect is direct or mediated by other factors. Researchers often combine multi-level modelling with epidemiological conceptual frameworks to analyse confounders at different levels of data clustering. Uthman and Ngnie-Teta et al. used both multi-level modelling and conceptual frameworks. The first to analyse factors associated with child malnutrition in Nigeria and the second to analyse the individual and community variables associated with the severity of anaemia in children in Mali and Benin [6,11]. Merlo et al. had extensively discussed the advantages and limitations of multi-level analysis in social epidemiology in dichotomous dependent variables [12,13]. Among the advantages of using multilevel regression discussed by Merlo are that this technique provides additional understanding of the distribution and determinants of geographical, social and individual disparities in health status.

In our analysis, we adopt this multi-level approach to explore the key risk factors, malnutrition and infections, in childhood anaemia globally adding a detailed analysis on the role of the consumption of different food groups.

## 2. Materials and Methods

### 2.1. Study Question

The primary objective of this analysis is to understand the relative importance of the countries’ human development, the geographical regions, community, household, maternal, child and nutritional variables in relation to the prevalence of anaemia between the age of 6 and 23 months. We have a special interest to explore how anaemia can be associated to the intake of different food groups as, both Global Alliance for Improved Nutrition (GAIN) and the Scaling Up Nutrition (SUN) framework acknowledge the importance of iron fortification of foods in reducing the risk of anaemia [14,15,16].

### 2.2. Data and Study Population

We used publicly available data from the Standard Demographic Health Survey (DHS) [USAID http://dhsprogram.com/] that was collected from 2005 to 2016 in Asia, North-Africa, the Middle East, Sub-Saharan Africa and Latin America. We originally identified 104 accessible standard DHS in the period of interest. However, we only kept the surveys that had information on haemoglobin and the covariates of interest. The final sample consisted of 52 surveys from 41 countries with over 136 thousand children from six to 23 months of age. The surveys included in this analysis are listed in Table S1 in the supplementary materials. DHS are national representative household surveys with primary focus on women 15 to 49 years old and children under 5 years old. The surveys had information about the health of each woman and her children as well as demographics and socioeconomics. Importantly, the DHS also includes a section on all foods that the woman’s youngest child was given in the past 24 h.

### 2.3. Study Variables

The outcome variable was anaemia defined by haemoglobin concentration adjusted by altitude using the anaemia threshold proposed by WHO for infants that is below 110 g/L [17]. We decided to use anaemia as a dichotomous variable instead of using a continuous haemoglobin concentration because odds ratios are directly interpretable. DHS collects haemoglobin data using the HemoCue system, which consists of a device that estimate haemoglobin concentrations from blood samples obtained in the field using finger prick (or heel prick) and yields results comparable to those obtained from other test systems [18].

**Figure 1:**
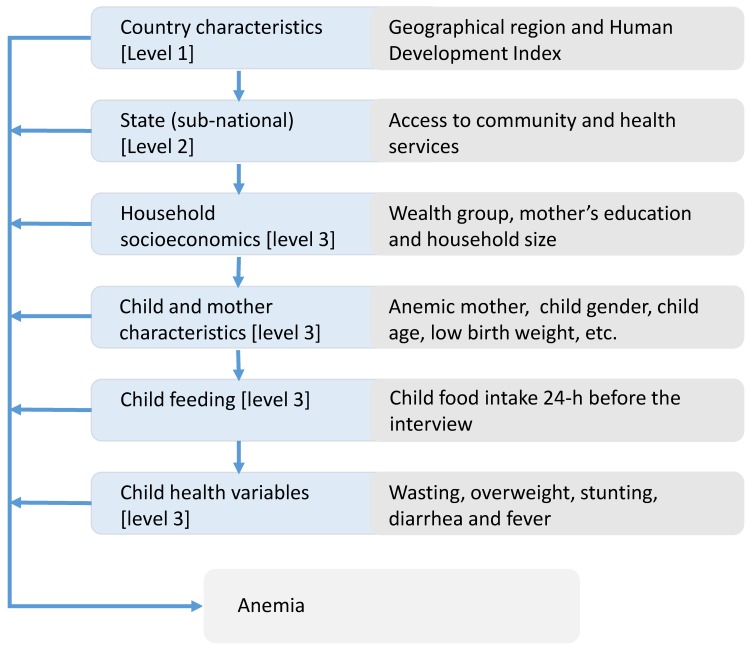
The hierarchy of the determinants of infant anaemia—conceptual framework for the multilevel analysis.

Figure 1 presents the conceptual framework used in the analysis to describe the hierarchical and inter-related nature of the various determinants contributing to infant anaemia. The framework proposed here extends the one used by Ngnie-Teta et al. The framework considers the development level of the geographic region because of the global nature of the analysis, as well as a better understanding of the potential role of the food intake [6]. Food intake from cross-sectional data can only be analysed in relation to health outcomes with large sample sizes, as there are many limitations in a cross-sectional data structure that are only partially overcome with larger samples. DHS only provides food intake information from a 24-h feeding. The arrows indicate in a simplified way how a group of determinants can influence other group variables.

We considered a three-level hierarchical model in our analysis. The first level corresponds to country characteristics that include the Human Development Index (HDI) classification at the time of the survey and the world region. Level 2 encompasses variables that classify sub-national entities (e.g., state or province) within a country based on the percentage of urban population and availability of health and community services. We measure accessibility to health care services as the percentage of infants’ mothers that had at least one antenatal visit during pregnancy and infants who had undergone a medical check-up before the 2 months of age. Accessibility to community services based on the percentage of household with electricity and piped water.

The Level 3, individual variables were classified into four groups: “household socioeconomic variables,” “infant and mother individual variables,” “Food intake variables” and “infant’s health status variables.”

Household socioeconomic variables included wealth index based on housing conditions and household’s assets, the mother’s level of education and household size parameters. We created dichotomous variables to distinguish households where there were six or more residents and another variable to identify households with three children or more under the age of five. Mother and child indicators included mother’s age at first birth (young mother first pregnancy at age <18 years yes/no), maternal anaemia, mother’s short stature (<150 cm) [19], child age, split into three-monthly sub-groups as 6–8, 9–11, 12–14, 15–17, 18–20, 21–23 months, low birth weight (yes/no) and gender (female).

The food groups generated in this analysis are similar to those used by Patel et al. [20]. However, we did not pool iron-rich food categories with the equivalent food categories that were not iron-rich, because, based on the systematic review of Eichler et al. [15], we wanted to check the effectiveness of fortifying food. Lutter et al. [21] explicitly point to the positive benefits of fortified complementary feeding. Child feeding variables corresponds to the assessment of the consumption of 11 food groups by the child in the 24 h before the interview. The 11 food-groups in the analysis are “breast milk,” “fortified milks,” “other milks,” “fortified baby food,” “foods made out of grains,” “potatoes & other tubers,” “fruits & vegetables,” “meat, poultry, fish, eggs,” “dried beans, peas, lentils, nuts,” “other dairy products” and “other solid-semisolid food.” The food groups correspond to the food categories asked in the standard DHS questioner questionnaire in rounds 5 and 6. In some surveys, there are more granularity on the food grouping. However, we collapsed the food groups to be consistent to surveys with a more generic food grouping in the sample. An alternative to the assessment of the different food groups, is the use of dietary diversity index. We have added a regression using the minimum dietary diversity score variable, with continued breastfeeding and a dummy variable to indicate consumption of fortified foods (Table S3 column 6 in the supplementary materials) and compared to the regression using 11 food groups.

The child health and nutritional indicators are: growth retardation defined as height for age z-score (HAZ) lower than minus two standard deviation (<−2SD), wasting defined as weight for height z-score (WHZ) < −2SD and overweight defined as WHZ > +2SD [22]. In addition, number of fever and diarrhoea cases during the two weeks previous to survey interview were included.

### 2.4. Data Analysis Method

The inclusion criteria for the explanatory variables in the analysis were based on previous studies on child anaemia and the availability of the variables in the DHS surveys. To verify the statistical relevance of the selected variables before inclusion in the multivariate models we ran univariate regression. Variables that were not significantly associated with anaemia or were not reported in a comparable way across surveys were excluded from the subsequent regression analysis.

In this global analysis, we estimated the odds ratio between anaemia in children 6 to 23 months old and the explanatory variables from three different procedures. First, from independent simple logistic regressions between anaemia and each explanatory variable. Second, from a multivariate traditional logistic regression with all individual level variables and using fixed effects for upper level factors. Third, from a multi-level logistic regression model. The multi-level model incorporated the explanatory variables grouped at the different levels of the hierarchy in line with the conceptual framework (Figure 1).

We used DHS primary sampling unites (PSUs) clustering, stratification and population weights to estimate robust standard errors to determine the p values. We also modelled the community effect as fixed effects in the TLR estimation while as a random effect in the MLR to obtain the estimates of higher-level coefficients, national and sub-national. We also ran regressions with and without population weights to check the sensitivity of the estimators to the survey weights. For all statistical estimations, we used Stata 13 (StataCorp. 2013. Stata Statistical Software: Release 13, StataCorp LP College Station, TX, USA).

The random effect multi-level analysis allowed us to estimate variance partition coefficients (VPC) from which we estimated the inter class correlation (ICC). The ICC allows us to estimate how much of the variation is explained by the heterogeneity among the clusters [23]. A high ICC would reflect a high clustering of anaemia prevalence at the national and subnational levels. We ran seven models to compare the changes on the ICC from adding additional groups of variables. The first model, the model 0 or null model, we only model the variance level structure specifying the levels without including any type of explanatory variables. In each subsequent model, from model 1 to model 6, we added gradually an additional group of explanatory variables as described above. Additionally, we estimated the incremental explanatory power added to the regression based on log-likelihood function to estimate the relative contribution of the individual level group of variables.

## 3. Results

The prevalence of anaemia in our sample of 136,024 children 6 to 23 months old was 70%, with the highest prevalence of 76% in the Sub-Saharan Africa and the lowest prevalence of 45% in the North Africa and Middle East surveys. The Latin American surveys registered an anaemia prevalence of 59% and the Asian surveys 70%. We present further descriptive data of our sample in Table S2. We ran likelihood ratio to test for heterogeneity among different surveys groups. Geographical differences were the most important source of heterogeneity. The likelihood ratio test (see the Table S5 in the supplementary materials) rejected the null hypothesis of homogeneity of the coefficients across geographical regions. However, the regression coefficients across different regions (see Table S3 in the supplementary materials) pointed out in the same direction. Thus, we can conclude that although there are significant differences, the direction of the association factors with anaemia is similar across the regions.

### 3.1. Multivariate and Multilevel Models

In Table 1, we display estimated odds ratios with a 95% confidence intervals (CI) and *p*-values from traditional logistic regression (TLR, multivariate) and the multi-level logistic regression (MLR). Because of the large sample size in individual level variables, we also could consider lower *p*-values threshold than the standard 0.05 for statistical significance, thus we provided in the Table 1 the *p*-values censored at 0.001. We could not include national and subnational variables in the TLR estimation due to collinearity as country and at state (subnational administrative divisions), heterogeneity is modelled with fixed effects. In contrast, in the MLR estimation, we were able to include country and state variables because the clustering effect is assumed to be random. We described in the following text the results of the MLR model. In the next section, we discuss the difference between both estimates.

At the country level variables, we found that children living in countries with higher HDI (above the 0.55 threshold for lower-middle class) had 29% lower odds of being anaemic than those living in less developed countries and remains statistically significant. By region, no significant difference was found once we controlled for all covariates based on 52 groups (surveys). Subnational level variables urbanity, access to health care services and access to community services failed to have a statistical significant estimator once considering all covariates.

Most of the individual level variables remained significant after taking into account all covariates. In the household socioeconomic variables group, wealth index was strongly associated with child anaemia. Children born in households at the fifth wealth quintile had a 27% lower odds of being anaemic compared with the peers in the first quintile (OR 0.73, 95% CI: 0.69 -0.76). In the same direction, children from mother mothers with secondary education or higher (OR 0.82, 95% CI: 0.78–0.85) and living in household with three or less children (OR 1.07, 95% CI: 1.03–1.11) were significantly less likely to be anaemic. For mother and infant variables, we found that maternal anaemia (OR 1.69, 95% CI: 1.65–1.74) and child low birth weight (OR 1.16, 95% CI: 1.12–1.19) were associated with higher anaemia rates. Girls (OR 0.89, 95% CI: 0.86–0.91) were less likely to be anaemic than boys. Anaemia and age had an inverted U pattern from six to 23 months of age. Anaemia was significantly higher in children from 12 to 14 months old (OR 1.07, 95% CI: 1.02–1.11) while significantly lower at 21 to 23 months of age (OR 0.74, 95% CI: 0.70–0.77) compared to the base line group from six to eight months of age. In the feeding variables groups, only fortified milks (OR 0.86, 95% CI: 0.82–0.90), fortified baby food (OR 0.90, 95% CI: 0.87–0.94) and tubers (OR 0.96, 95% CI: 0.93–0.98) were significantly associated with lower anaemia rates. Consumption of foods made from grains, mainly in form of homemade porridge, bread or noodles was associated with higher anaemia rates (OR 1.09, 95% CI: 1.05–1.12). Breastmilk was associated with higher anaemia rates (OR 1.07, 95% CI: 1.04–1.11) in the multilevel regression but it was not significant in the traditional logistic regression. Children’s health variables were significantly associated with higher anaemia rates: wasting (OR 1.08, 95% CI: 1.04–1.12), stunting (OR 1.20, 95% CI: 1.16–1.23), diarrhoea (OR 1.05, 95% CI: 1.01–1.08) and fever (OR 1.09, 95% CI: 1.06–1.13).

### 3.2. Interclass Correlation and Explained Variance

**Figure 2:**
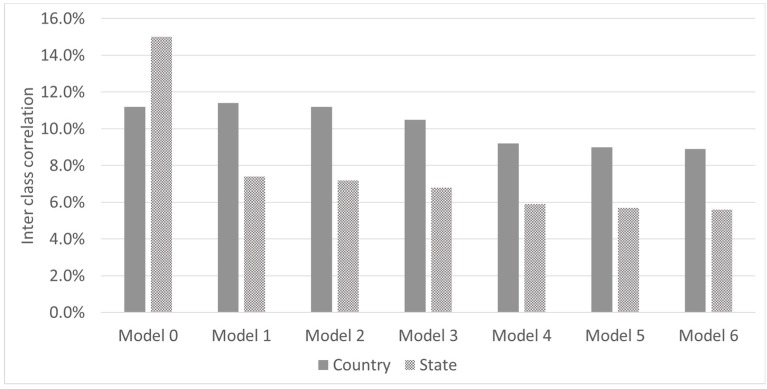
Inter class correlation by models. Model 0: Null model only variance level cluster; Model 1: adds national level variables; Model 2: adds state (subnational) level variables; Model 3: adds household socioeconomic variables; Model 4: adds child and mother characteristics; Model 5: adds feeding variables; Model 6: adds child health variables.

In the multi-level analysis, we estimated the interclass correlation and the within country variability (community) as we introduce the different levels in the multivariate analysis. Figure 2 shows that 11% and 15% of the variability is attributable to differences between countries and states (or other subnational administrative division among countries). The interclass correlations decrease as additional groups of variables are introduced into the model. In the full model, the interclass correlation stands at 9% and 5.7% percent for the national and subnational level respectively.

**Figure 3:**
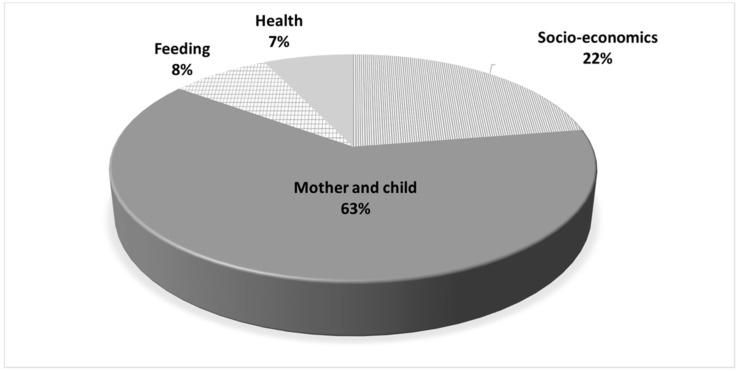
Contribution of individual level variables group to the incremental explanatory power of the regression (Pseudo R2).

In Figure 3, we present the contribution of individual level variables to the total explanatory power of the model. The mother and child variable groups contributed 63% of the incremental pseudo R2. Socioeconomic variables accounted for 22% of the incremental explanatory power of the model whereas feeding variables and health variables contributed 8% and 7% respectively.

## 4. Discussion

This research pools data from DHS undertaken in Asia, Middle East, North Africa, Sub-Saharan Africa and Latin America. This is the first study that combines over 50 national surveys in low-and-lower-middle-income countries with a sample size surpassing 130 thousand children to shed some light on the association between anaemia and related factors using MLR and TLR regression techniques. We found that the estimations with both approaches were very similar for all individual level variables except for the feeding variables group. In the feeding variables group, some of the coefficients that were significant under the MLR were not significant at the TLR or vice versa, although the associations were consistently in the same direction. Ngnie-Teta et al. did a similar comparison on MLR and TLR using DHS from Benin and Mali and did not find major differences among the estimated coefficients using either approach [6]. One possible explanation for these differences could be due to the nature of these variables. The effect of food types could partially be indirect (intermediate variables). Our estimates of the interclass correlations, once we control by full covariates, were relatively low, 9% at national level and 5.7% at subnational level.

As expected, higher level of HDI were significantly associated with lower prevalence of anaemia even after controlling for other variables. State level variables were not statistically significant after controlling for the full variables of the model. This may indicate that community variables probably are also reflected in socioeconomic and other individual variables thus their statistical power is diluted. Household socioeconomic variables are good predictors of the likelihood of anaemia in children at this age in line with previous literature [24]. Child and mother variables had the highest incremental explanatory power with maternal anaemia coefficient being the greatest in magnitude. The strong association between maternal and child anaemia could be due to many factors that determine anaemia that are shared between mother and child [24,25]. Age groups coefficients were statistically significant suggesting an inverted-U-pattern between the prevalence of anaemia and age in months. [26,27,28].

Few coefficients from the feeding variable groups were statistically significant. Nonetheless, among the food groups fortified milks and fortified baby foods had the strongest significant association with lower anaemia rates as there is a biochemical pathway supported by several clinical trials and systematic reviews [15,16]. Cross-association between feeding groups and health outcomes such as anaemia may reflect a causal relationship between nutrient density content of the foods and the specific impact on health outcome [27]. Nonetheless, because food consumption was assessed during the 24h preceding the haemoglobin measurement, a significant association could be pointing at persistent feeding habits. Randomized Clinical Trials (RCTs) have shown a positive effect of iron fortified products on haemoglobin after 4 to 6 months of regular consumption [15,29]. Therefore, a contemporaneous association between feeding habits and haemoglobin or anaemia requires that current and past consumption patterns to be highly correlated to potentially reflect the possible causal path on nutrient density of the foods and health outcomes [30]. Food made out of grains, including homemade porridges (no fortified), bread and noodles, had a statistical significantly positive association with anaemia. This association could arise because these foods could replace other iron-rich foods in the diet or the presence of phytic acids that reduce the absorption of iron [31]. Breastmilk had no clear association with anaemia, in the TLR the association was not significant while it was negatively associated with anaemia in MLR. Breastmilk contains very little amounts of iron and if the mother’s diet is not diversified breastmilk would not contribute enough to meet the infants daily iron requirements [32,33,34,35]. However, breastmilk prevents infections that could cause anaemia [34]. Beyond anaemia, a longer duration of breastfeeding brings many health benefits to the child [36]. After the first six months of life where children should be exclusively breast fed, continuous breastfeeding along with the introduction of complementary feeding should provide the nutrients and vitamins needed for the child’s development. Alternative to associating different food groups with anaemia, we also ran a regression with three feeding variables only (whether the child is breastfed, met the minimum dietary diversity and if was fed with fortified foods). The results are shown on Table S3 column 6 in the supplementary materials. In our sample dietary diversification of children is low (see Graph S1 in the supplementary materials), we conclude that both meeting the minimum dietary diversity (OR 0.89, 95% CI: 0.84–0.94) and the consumption of fortified foods (OR 0.84, 95% CI 0.79–0.89) are associated with lower anaemia in infancy. These results are consistent with the endorsement Global Alliance for Improved Nutrition (GAIN) [37] of fortified infant cereals as a vehicle to provide infants with vitamins and minerals to fight micronutrient deficiencies. It is also consistent with the report of the Scale Up nutrition report from the World Bank “Complementary and therapeutic feeding interventions that provide micronutrient-fortified and/or -enhanced complementary foods for the prevention and treatment of moderate malnutrition among children 6–23 months of age” as cost-effective public health interventions [38].

The associations between anaemia and health variables are in the expected direction according to the previous literature [6,25]. Wasting and stunting were significantly associated with higher anaemia prevalence. Similar to anaemia, wasting and stunting could be related to long-term insufficient nutrient intake and frequent infections. The inclusion of wasting and stunting may reduce the omitted variable bias on other parameters as we take indirectly into consideration environmental factors and dietary diversity in the past. Fever and diarrhoea result frequently from infectious diseases such as malaria. In the absence of malaria-controlling variables in this study such as bed nets and malaria testing these variables could improve the estimates.

We want to make the reader aware not interpret the estimated ORs as a Risk Ratios (RRs) because the prevalence of anaemia in our sample is high. Using the ORs as an approximation to RRs, when the outcome variable is common (>10%), inflates the size of the association [39]. Barros and Hirakata 2003 [40] recommend reporting RRs from Cox/Poisson regression models instead of logistic regressions ORs in cross-sectional analysis as the RRs are easier to interpret and communicate to non-specialist. Nevertheless, ORs have many advantages, the logistic estimation is computationally more efficient and reporting ORs in common outcomes is correct as long as the researcher properly interprets the magnitude of the association [41]. Cook et al. 2002 recommends usage of ORs in common outcomes and alerting the reader on the proper interpretation [42]. For a comprehensive discussion on understanding and use of RRs and OR, we refer to Wilber and Fu [43]. In this regard, we provide in Table S4 in the supplementary materials, a comparison on the estimation of the RR and OR using the STATA’s adjrr command for estimating the RR. The results are always in same direction with difference size effect.

This study has a number of limitations. First, using cross-sectional data prevented us to do any causal inference. As discussed above, some associations reflect a causal pathway but we do not have the appropriate data structure to support this. Second, our data are from various geographies, therefore our estimates represent an average of our sample that may differ between countries in the sample. However, our estimates are aligned with the previous findings in the literature and highlight future areas of interest for additional studies. Increasing our sample by increasing the total number of surveys came at a cost of limiting the number of variables analysed such as excluding measures of bed nets or prevalence of malaria. Although few DHS have information on this, most DHS focus more on other child and maternal aspects. Last yet important, we did not have information on the causes of anaemia as we lack additional biomarkers.

## 5. Conclusions

The prevalence of anaemia during the complementary feeding in low and lower-middle-income countries remains high. This is a critical period in life as the anaemia during this age could lead to improper child neurodevelopment. Around two thirds of the children at six months of age in our sample were anaemic, early life interventions should prioritize reducing the likelihood of being anaemic at that age. Improving overall household living conditions as well as increasing maternal education, delaying and spacing childbearing and introducing iron rich foods at the beginning of the complementary feeding period [44] could also have a positive impact on reducing anaemia in infancy. Public health programs should target infants from anaemic mothers and infants in deprived households as they are at higher risk of anaemia. Given the association between fever and diarrhoea with anaemia, reducing infection diseases, through sanitation, vaccination and malaria prevention could enhance haemoglobin concentrations.

## Figures and Tables

**Table 1:** Estimated odds ratios traditional logistic and multilevel logistic regressions on anaemia in children 6 to 23 months old.

	Adjusted TLR OR(95% CI)	*p*	Adjusted MLR OR(95% CI)	*p*
***Country level factors***				
HDI at the time of the survey				
*Low*			1	
*Lower middle/ middle*			0.71 (0.50–0.99)	0.045
World region				
*Asia*			1	
*North Africa and Middle East*			0.52 (0.25–1.11)	0.090
*Sub-Saharan Africa*			1.35 (0.94–1.93)	0.099
*Latin America*			0.89 (0.55–1.42)	0.620
***Sub-national level factors***				
Type of residence				
*Rural*			1	
*Urban*			1.15 (0.96–1.38)	0.122
Access to health services				
*Low*			1	
*High*			0.94 (0.83–1.07)	0.368
*Access to community services*				
*Low*			1	
*High*			0.92 (0.80–1.05)	0.222
***Socio-economic variables***				
Wealth quintile				
*Lowest*	1		1	
*Lower*	0.92 (0.86–0.99)	0.019	0.95 (0.92–0.99)	0.011
*Middle*	0.91 (0.84–0.99)	0.027	0.91 (0.88–0.95)	<0.001
*High*	0.88 (0.79–0.98)	0.016	0.85 (0.81–0.89)	<0.001
*Highest*	0.80 (0.70–0.91)	<0.001	0.73 (0.69–0.76)	<0.001
Household size				
*Less than 6 members*	1		1	
*6 or more members*	1.03 (1.00–1.06)	0.042	1.00 (0.97–1.02)	0.826
Children under 5				
*3 or less*	1		1	
*More than 3*	1.09 (1.05–1.14)	<0.001	1.07 (1.03–1.11)	<0.001
Mother education				
*None*	1		1	
*Primary*	0.86 (0.82–0.91)	<0.001	0.91 (0.88–0.95)	<0.001
*Secondary and above*	0.74 (0.70–0.80)	<0.001	0.82 (0.78–0.85)	<0.001
***Mother and child variables***				
Mother’s age at first birth				
*18 years old and older*	1		1	
*Younger than 18 years old*	1.05 (1.01–1.10)	0.021	1.06 (1.02–1.10)	<0.001
Mother with anaemia				
*No*	1		1	
*Yes*	1.78 (1.68–1.89)	<0.001	1.69 (1.65–1.74)	<0.001
Stunted mother				
*No*	1		1	
*Yes*	1.00 (0.96–1.04)	0.897	1.02 (0.99–1.05)	0.147
Child’s low birth weight				
*No*	1		1	
*Yes*	1.20 (1.15–1.26)	<0.001	1.16 (1.12–1.19)	<0.001
Child’s gender				
*Boy*	1		1	
*Girl*	0.89 (0.86–0.92)	<0.001	0.89 (0.86–0.91)	<0.001
Child’s age				
*6 to 8 months*	1		1	
*9 to 11 months*	1.06 (1.00–1.12)	0.061	1.06 (1.01–1.10)	0.015
*12 to 14 months*	1.06 (0.99–1.14)	0.075	1.07 (1.02–1.11)	0.005
*15 to 17 months*	0.98 (0.88–1.10)	0.778	0.99 (0.94–1.03)	0.563
*18 to 20 months*	0.88 (0.78–1.00)	0.045	0.87 (0.83–0.91)	<0.001
*21 to 23 months*	0.74 (0.64–0.86)	<0.001	0.74 (0.70–0.77)	<0.001
Child’s birth order				
*Second or later*	1		1	
*First child*	1.03 (0.99–1.06)	0.149	1.02 (0.99–1.05)	0.231
***Child feeding variables***				
Breast milk				
*No*	1		1	
*Yes*	1.05 (0.98–1.12)	0.170	1.07 (1.04–1.11)	<0.001
Fortified milks				
*No*	1		1	
*Yes*	0.83 (0.78–0.89)	<0.001	0.86 (0.82–0.90)	<0.001
Other milks				
*No*	1		1	
*Yes*	1.09 (1.03–1.16)	0.003	1.00 (0.97–1.03)	0.965
Fortified baby food				
*No*	1		1	
*Yes*	0.86 (0.81–0.92)	<0.001	0.90 (0.87–0.94)	<0.001
Foods made from grains				
*No*	1		1	
*Yes*	1.10 (1.05–1.16)	<0.001	1.09 (1.05–1.12)	<0.001
Potatoes & other tubers				
*No*	1		1	
*Yes*	0.94 (0.90–0.99)	0.009	0.96 (0.93–0.98)	0.002
Meat, poultry, fish, eggs				
*No*	1		1	
*Yes*	0.91 (0.84–0.98)	0.011	0.99 (0.96–1.03)	0.756
Fruits and vegetables				
*No*	1		1	
*Yes*	0.97 (0.93–1.00)	0.077	1.00 (0.97–1.03)	0.825
Dried beans, peas and nuts				
*No*	1		1	
*Yes*	0.97 (0.92–1.01)	0.148	1.02 (0.98–1.05)	0.410
Other dairy products				
*No*	1		1	
*Yes*	1.08 (1.00–1.15)	0.040	1.02 (0.98–1.05)	0.410
Other solid-semisolids foods				
*No*	1		1	
*Yes*	0.92 (0.88–0.96)	<0.001	0.99 (0.96–1.02)	0.688
***Child’s health variables***				
Wasting (WHZ<-2SD)				
*No*	1		1	
*Yes*	1.15 (1.09–1.22)	<0.001	1.08 (1.04–1.12)	<0.001
Overweight (WHZ>+2SD)				
*No*	1		1	
*Yes*	0.83 (0.77–0.90)	<0.001	0.84 (0.78–0.90)	<0.001
Stunting (HAZ<-2SD)				
*No*	1		1	
*Yes*	1.23 (1.18–1.28)	<0.001	1.20 (1.16–1.23)	<0.001
Diarrhoea in last two weeks				
*No*	1		1	
*Yes*	1.08 (1.03–1.13)	0.001	1.05 (1.01–1.08)	0.007
Fever in last two weeks				
*No*	1		1	
*Yes*	1.11 (1.07–1.16)	<0.001	1.09 (1.06–1.13)	<0.001

Note: Each feeding variable indicates whether the infant was exposed or not to that food type. For instance, breast milk refers to any breast milk independently of any other complementary food.

## Supplementary Materials

The following are available online at http://www.mdpi.com/2072-6643/10/9/1269/s1, Table S1: Countries and years in sample, Table S2: Descriptive statistics of the sample, Table S3: Traditional Logistic Regression by world regions and comparison between individual food and minimum dietary diversity regressions, Table S4: Comparison between the Odds Ratio (OR) estimated from a logistic regression and the Risk Ratio (RR) estimated using the Delta Method approximation from the logistic regression. Table S5: Likelihood ratio test to verify the homogeneity assumption across geographical regions. Graph S1: Dietary diversity by age in months. Abbreviation: DDS: Dietary Diversity Score.
