# Supplementary material for: Association between Anaemia in Children 6 to 23 Months Old and Child, Mother, Household and Feeding Indicators
Source: Nutrients. 2018 Sep 8;10(9):1269. doi: 10.3390/nu10091269 (PMC6163758; doi:10.3390/nu10091269)
Supplement: Supplementary file 1 [file nutrients-10-01269-s001.pdf]

Supplementary

Table S1. Countries and years in sample.

World region	Country and survey year in parenthesis
Asia (11 surveys)	Azerbaijan (2006); Bangladesh (2011); Cambodia (2010, 2014); India (2005, 2015); Kyrgyzstan (2012); Myanmar (2015); Nepal (2006, 2011); Timor-Leste (2009)
North-Africa & Middle-East (2 surveys)	Egypt (2014); Jordan (2012)
Sub-Saharan Africa (33 surveys)	Benin (2006, 2011); Burkina Faso (2010); Burundi (2010); Cameroon (2011); Congo (2011); Democratic Republic of the Congo (2013); Cote d'Ivoire (2011); Ethiopia (2011); Gabon (2012); Gambia (2013); Ghana (2014); Guinea (2012); Lesotho (2014); Malawi (2010, 2015); Mali (2012); Mozambique (2011); Namibia (2013); Niger (2012); Rwanda (2010, 2014); São Tomé and Príncipe (2008); Senegal (2010); Sierra Leone (2008, 2013); Swaziland (2006); Tanzania (2015); Togo (2013); Uganda (2006); Zimbabwe (2005, 2010, 2015)
Latin-America (6 surveys)	Guatemala (2014); Guyana (2009); Haiti (2005, 2012); Honduras (2005, 2011)

Table S2. Descriptive statistics of the sample.

	Asia	North Africa and Middle East	Sub-Saharan Africa	Latin America	Total
**Dependent variable**					
Anemia	70.1%	44.9%	75.6%	58.6%	70.2%
**Country and community variables**					
HDI at the time of the survey	81.9%	100.0%	9.7%	81.9%	61.5%
Mostly urban state	3.9%	74.6%	12.0%	21.1%	9.4%
Access to community services	53.7%	100.0%	6.3%	71.6%	42.6%
Access to health care	41.9%	100.0%	77.8%	92.7%	57.9%
	Asia	North Africa and Middle East	Sub-Saharan Africa	Latin America	Total
**Household, mother and child variables**					
Household size					
6 or more members	57.9%	43.8%	58.%	54.2%	57.3%
More than 3 children U5	16.4%	18.3%	24.8%	15.5%	18.8%
Maternal education					
No education	28.8%	7.9%	36.3%	13.6%	29.2%
Primary education	15.5%	6.9%	37.5%	57.%	25.2%
Secondary or higher	55.7%	85.2%	26.2%	29.4%	45.7%
Mother younger than 18 years old	12.8%	8.%	33.%	33.4%	20.3%
Mother with anemia	55.6%	30.6%	38.3%	21.9%	47.2%
Low birth weight child	31.4%	20.9%	17.3%	19.5%	26.1%
Female child	47.9%	47.4%	50.%	48.6%	48.5%
First born child	36.3%	24.9%	22.4%	31.9%	31.7%

**Child feeding variables**					
Breast milk	84.8%	52.8%	82.1%	74.1%	82.3%
Fortified milks	9.8%	5.7%	5.7%	9.5%	8.5%
Other milks	39.7%	49.5%	15.6%	33.8%	32.5%
Fortified baby food	14.4%	7.6%	9.5%	27.8%	13.9%
Foods made from grains	68.3%	73.9%	65.5%	81.8%	68.8%
Potatoes & other tubers	32.%	44.9%	32.6%	46.%	33.6%
Meat, poultry, fish, eggs	20.7%	57.6%	41.7%	66.2%	31.5%
Fruits and vegetables	43.3%	58.9%	49.3%	55.4%	46.4%
Dried beans, peas and nuts	15.1%	18.2%	21.9%	58.1%	20.8%
Other dairy products	15.5%	67.3%	9.1%	34.9%	16.5%
Other solid-semisolid foods	25.%	45.5%	42.7%	54.3%	33.%
**Child's health variables**					
Wasting (WHZ<-2SD)	21.8%	7.2%	11.2%	3.3%	16.9%
Overweight (WHZ>+2SD)	6.3%	19.3%	11.7%	14.7%	8.9%
Stunting (HAZ<-2SD)	34.4%	12.5%	29.6%	27.7%	31.9%
Diarrhea in last two weeks	15.1%	24.5%	25.8%	29.9%	19.6%
Fever in last two weeks	18.8%	27.%	27.4%	29.7%	22.4%
Number of observations	82 343	3 231	39 440	11 529	136 543

Table S3. Traditional Logistic Regression by world regions and comparison between individual food and minimum dietary diversity regressions.

VARIABLES	Asia	North Africa and Middle East	Sub-Saharan Africa	Latin America	All	All with the DDS
**Socio-economic variables**						
Wealth quintile [lowest]						
Lower	0.89**	0.95	0.98	1.05	0.92**	0.93**
	(0.80:0.98)	(0.81:1.11)	(0.90:1.06)	(0.92:1.20)	(0.86:0.99)	(0.87:0.99)
Middle	0.88**	0.75*	0.95	1.07	0.91**	0.91**
	(0.77:1.00)	(0.56:1.01)	(0.87:1.03)	(0.91:1.25)	(0.84:0.98)	(0.84:0.99)
High	0.83**	0.81	0.95	0.95	0.88**	0.88**
	(0.70:0.98)	(0.63:1.05)	(0.87:1.04)	(0.80:1.13)	(0.79:0.97)	(0.79:0.98)
Highest	0.79**	0.56***	0.80***	0.71***	0.80***	0.81***
	(0.65:0.96)	(0.39:0.79)	(0.72:0.89)	(0.58:0.87)	(0.70:0.91)	(0.71:0.93)
Household size						
6 or more members	1.03	1.13	1.01	1.02	1.03*	1.03**
	(0.99:1.07)	(0.92:1.38)	(0.96:1.06)	(0.93:1.13)	(1.00:1.06)	(1.00:1.06)
More than 3 children under 5	1.04	1.04	1.17***	1.06	1.09***	1.08***
	(0.98:1.09)	(0.87:1.24)	(1.09:1.26)	(0.95:1.19)	(1.05:1.14)	(1.04:1.13)
Mother education [none]						
Primary education	0.85***	1.15	0.86***	0.98	0.86***	0.86***
	(0.79:0.92)	(0.77:1.70)	(0.79:0.93)	(0.89:1.07)	(0.82:0.91)	(0.82:0.91)
Secondary or higher	0.74***	0.98	0.78***	0.85**	0.74***	0.74***
	(0.67:0.81)	(0.65:1.49)	(0.71:0.86)	(0.75:0.98)	(0.70:0.80)	(0.69:0.80)
**Mother and child variables**						
Young mother (18y<)	1.01	1.33**	1.10***	1.07*	1.05**	1.06**
	(0.94:1.09)	(1.06:1.69)	(1.04:1.17)	(0.99:1.17)	(1.01:1.11)	(1.01:1.10)
Anemic mother	1.85***	1.29***	1.71***	1.61***	1.78***	1.79***
	(1.71:2.01)	(1.08:1.53)	(1.60:1.82)	(1.45:1.79)	(1.68:1.89)	(1.68:1.90)
Stunted mother	1	1.16	1.01	0.94	1	0.99
	(0.96:1.05)	(0.84:1.61)	(0.93:1.09)	(0.85:1.05)	(0.96:1.04)	(0.95:1.03)
Low birth weight	1.23***	1.25***	1.16***	1.04	1.20***	1.21***
	(1.16:1.30)	(1.07:1.47)	(1.08:1.24)	(0.93:1.17)	(1.15:1.26)	(1.16:1.27)
Girl	0.91***	0.91	0.83***	0.88***	0.89***	0.88***
	(0.87:0.95)	(0.74:1.12)	(0.78:0.87)	(0.81:0.94)	(0.86:0.92)	(0.85:0.91)
Age group [6 to 8m]						
9 to 11m	1.09**	0.83	1.06	0.86**	1.06*	1.06**
	(1.01:1.18)	(0.58:1.19)	(0.96:1.16)	(0.75:0.98)	(1.00:1.11)	(1.00:1.12)
12 to 14m	1.18***	0.88	0.94	0.72***	1.06	1.07**
	(1.08:1.28)	(0.63:1.22)	(0.85:1.04)	(0.64:0.82)	(0.98:1.14)	(1.00:1.15)
15 to 17m	1.15**	0.85**	0.83***	0.53***	0.98	0.99
	(1.01:1.31)	(0.73:0.99)	(0.74:0.93)	(0.46:0.61)	(0.88:1.10)	(0.89:1.11)
18 to 20m	1.08	0.71***	0.67***	0.46***	0.88**	0.89*
	(0.95:1.23)	(0.57:0.90)	(0.59:0.76)	(0.39:0.55)	(0.78:0.99)	(0.79:1.01)
21 to 23m	0.89	0.71***	0.62***	0.32***	0.74***	0.75***
	(0.75:1.07)	(0.58:0.86)	(0.55:0.71)	(0.27:0.38)	(0.63:0.86)	(0.65:0.86)
First born child	1	0.91	1.09***	1.04	1.03	1.02
	(0.96:1.05)	(0.75:1.10)	(1.03:1.16)	(0.95:1.15)	(0.99:1.06)	(0.99:1.06)
**Child feeding variables**						
Breast milk	0.94	1.22 *	1.11 ***	1.18 ***	1.05	1.05
	(0.85:1.04)	(0.98:1.51)	(1.03:1.21)	(1.06:1.32)	(0.98:1.12)	(0.97:1.13)
Fortified milks	0.83 ***	0.84	0.80 ***	0.86*	0.83 ***	
	(0.77:0.90)	(0.55:1.27)	(0.70:0.92)	(0.72:1.01)	(0.78:0.89)	
Other milks	1.09 ***	0.88	1.08 *	0.93	1.09 ***	
	(1.02:1.17)	(0.71:1.10)	(0.99:1.18)	(0.83:1.05)	(1.02:1.16)	

Fortified baby food	0.84 ***	0.84 *	0.89 **	0.94 *	0.86 ***	
	(0.78:0.92)	(0.69:1.03)	(0.81:0.98)	(0.86:1.02)	(0.81:0.92)	
Bread, rice, noodles	1.10 **	1.09	1.07 **	1.20 ***	1.10 ***	
	(1.02:1.18)	(0.86:1.37)	(1.01:1.13)	(1.07:1.34)	(1.05:1.16)	
Potatoes and other tubers	0.94 *	0.99	0.94**	0.89 ***	0.94 **	
	(0.88:1.01)	(0.85:1.16)	(0.88:0.99)	(0.82:0.96)	(0.91:0.99)	
Meat, poultry, fish, eggs	0.78 ***	0.91	1.13 ***	1.02	0.91***	
	(0.70:0.88)	(0.77:1.07)	(1.07:1.20)	(0.92:1.12)	(0.84:0.98)	
Fruits and vegetables	0.99	0.93	0.90 ***	0.99	0.97	
	(0.93:1.05)	(0.78:1.12)	(0.84:0.95)	(0.91:1.07)	(0.93:1.01)	
Beans, peas, lentils and nuts	0.97	1.15	0.94*	1.06	0.97	
	(0.90:1.06)	(0.96:1.38)	(0.88:1.00)	(0.97:1.16)	(0.92:1.01)	
Other dairy products	1.17 ***	1.07	0.96	0.98	1.08 **	
	(1.06:1.30)	(0.97:1.19)	(0.86:1.07)	(0.89:1.08)	(1.01:1.16)	
Other solid-semisolid food	0.88 ***	0.94	1.02	0.93	0.92 ***	
	(0.82:0.94)	(0.77:1.14)	(0.96:1.08)	(0.82:1.05)	(0.88:0.96)	
Dietary Diversity Score >3						0.89 ***
						(0.84:0.94)
Fortified foods						0.84 ***
						(0.79:0.89)
**Child's health variables**						
Wasting (WHZ<-2SD)	1.16 ***	0.68 *	1.17 ***	0.94	1.15 ***	1.15 ***
	(1.09:1.23)	(0.45:1.02)	(1.07:1.29)	(0.75:1.18)	(1.09:1.21)	(1.09:1.22)
Overweight (WHZ>+2SD)	0.87 **	0.95	0.76 ***	0.88	0.83 ***	0.83 ***
	(0.76:0.98)	(0.80:1.12)	(0.67:0.85)	(0.73:1.07)	(0.77:0.90)	(0.77:0.90)
Stunting (HAZ<-2SD)	1.26 ***	1.04	1.14 ***	1.23 ***	1.23***	1.23 ***
	(1.20:1.32)	(0.83:1.31)	(1.07:1.22)	(1.11:1.37)	(1.18:1.28)	(1.18:1.28)
Diarrhea in last two weeks	1.08 **	1.11	1.04	1.12 **	1.08 ***	1.08 ***
	(1.01:1.17)	(0.93:1.32)	(0.98:1.10)	(1.01:1.23)	(1.03:1.13)	(1.03:1.13)
Fever in last two weeks	1.08 ***	1.17	1.24 ***	0.98	1.11 ***	1.12 ***
	(1.03:1.14)	(0.93:1.48)	(1.16:1.33)	(0.86:1.12)	(1.07:1.16)	(1.07:1.16)

Note: Significance *** p<0.01, ** p<0.05, * p<0.1

Table S4. Comparison between the Odds Ratio (OR) estimated from a logistic regression and the Risk Ratio (RR) estimated using the Delta Method approximation from the logistic regression.

	OR	RR
**Socio-economics**		
Wealth quintile (Ref lowest)		
Lower	0.92 ** (0.86–0.99)	0.98 ** (0.96–1)
Middle	0.91 ** (0.84–0.98)	0.97 ** (0.95–0.99)
High	0.88 ** (0.79–0.97)	0.96 ** (0.93–0.99)
Highest	0.80 *** (0.70–0.91)	0.94 *** (0.9–0.97)
Household size		
6 or more members	1.03 * (1.00–1.06)	1.01 * (1–1.02)
More than 3 children under 5	1.09 *** (1.05–1.14)	1.02 *** (1.01–1.03)
Mother education		
Primary education	0.86 *** (0.82–0.91)	0.96 *** (0.95–0.98)
Secondary or higher	0.74 *** (0.70–0.80)	0.92 *** (0.9–0.94)
**Mother and child variables**		
Young mother (18y<)	1.05 ** (1.01–1.11)	1.02 *** (1–1.03)
Anemic mother	1.78 *** (1.68–1.89)	1.17 *** (1.15–1.2)
Stunted mother	1 (0.96–1.04)	1 (0.99–1.01)
Low birth weight	1.20 *** (1.15–1.26)	1.05 *** (1.04–1.06)
Girl	0.89 *** (0.86–0.92)	0.97 *** (0.96–0.98)
Age group [6 to 8m]		
9 to 11m	1.06 * (1.00–1.11)	1.01* (1–1.03)
12 to 14m	1.06 (0.98–1.14)	1.02 (1–1.03)
15 to 17m	0.98 (0.88–1.10)	0.99 (0.96–1.03)
18 to 20m	0.88 ** (0.78–0.99)	0.96 ** (0.93–1)
21 to 23m	0.74 *** (0.63–0.86)	0.91 *** (0.87–0.96)
First born child	1.03 (0.99–1.06)	1.01 (1–1.02)
**Child feeding variables**		
Breast milk	1.05 (0.98–1.12)	1.01 (0.99–1.03)
Fortified milks	0.83 *** (0.78–0.89)	0.95 *** (0.93–0.97)
Other milks	1.09 *** (1.02–1.16)	1.02 *** (1.01–1.04)
Fortified baby food	0.86 *** (0.81–0.92)	0.96 *** (0.94–0.98)
Bread, rice, noodles	1.10 *** (1.05–1.16)	1.03 *** (1.02–1.05)
Potatoes and other tubers	0.94 ** (0.91–0.99)	0.99 ** (0.97–1)
Meat, poultry, fish, eggs	0.91 *** (0.84–0.98)	0.97 *** (0.95–0.99)
Fruits and vegetables	0.97 (0.93–1.01)	0.99 (0.98–1)
Beans, peas, lentils and nuts	0.97 (0.92–1.01)	0.99 (0.98–1)
Other dairy products	1.08 ** (1.01–1.16)	1.02 ** (1–1.04)
Other solid-semisolid food	0.92 *** (0.88–0.96)	0.98 *** (0.96–0.99)
	OR	RR
**Child's health variables**		
Wasting (WHZ<-2SD)	1.15 *** (1.09–1.21)	1.04 *** (1.02–1.05)
Overweight (WHZ>+2SD)	0.83 *** (0.77–0.90)	0.95 *** (0.93–0.97)
Stunting (HAZ<-2SD)	1.23 *** (1.18–1.28)	1.06 *** (1.05–1.07)
Diarrhea in last two weeks	1.08 *** (1.03–1.13)	1.02 *** (1.01–1.03)
Fever in last two weeks	1.11 *** (1.07–1.16)	1.03 *** (1.02–1.04)

Note: Significance *** p<0.01, ** p<0.05, * p<0.1

Table S5: Likelihood ratio test to verify the homogeneity assumption across geographical regions

Assumption: (All) nested in (Asia, MENA, SSA, LA)

LR chi2(81) = 595.38; Prob > chi2 = 0.0000

Model	Obs	ll(null)	ll(model)	df	AIC	BIC
All	136 543	-80414.7	-73169.3	578	147494.5	153152.3
Asia	82 343	-48306.4	-44758.1	190	89896.2	91659.01
MENA	3 231	-2254.72	-2152	40	4384.002	4627.899
SSA	39 440	-21228.9	-19079.2	329	38816.48	41630.06
LA	11 529	-7535.92	-6882.24	100	13964.49	14696.17

Abbreviations: MENA: Middle East and North Africa; SSA: Sub-Saharan Africa; LA: Latin America; df: degrees of freedom; AIC: Akaike's information criterion; BIC Bayesian information criterion.

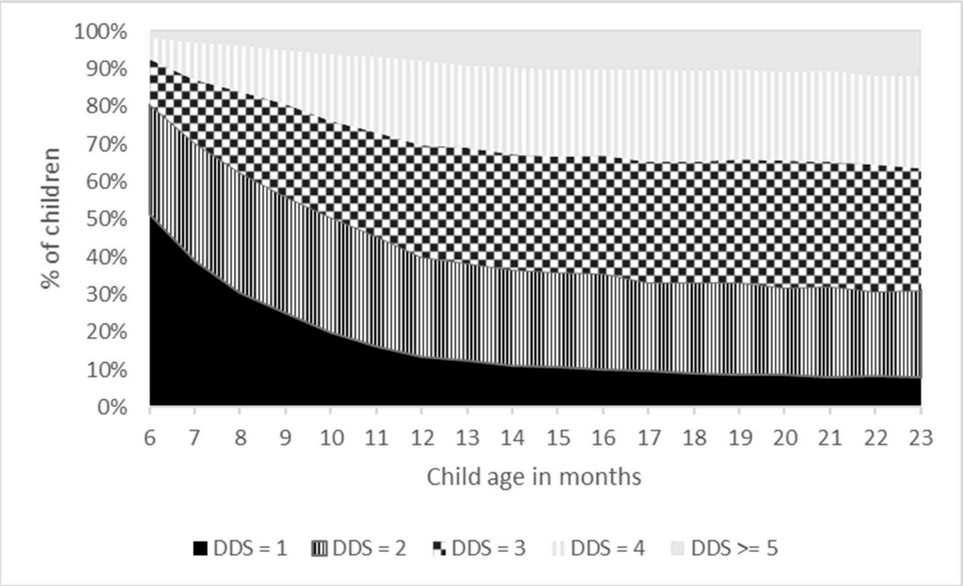

Graph S1. Dietary diversity by age in months. Abbreviation: DDS: Dietary Diversity Score.
